# Supplementary material for: Behavioral algorithms of ontogenetic switching in larval and juvenile zebrafish phototaxis
Source: iScience. 2026 Jun 5;29(6):116190. doi: 10.1016/j.isci.2026.116190 (PMC13253138; doi:10.1016/j.isci.2026.116190)
Supplement: Document S1. Figures S1–S7 [file mmc1.pdf]

iScience, Volume *29*

Supplemental information

Behavioral algorithms of ontogenetic switching in larval and juvenile zebrafish phototaxis

Maxim Q. Capelle, Katja Slangewal, Panagiotis E. Eleftheriadis, and Armin Bahl

SUPPLEMENTAL INFORMATION

Document S1

Figures S1 to **S7**

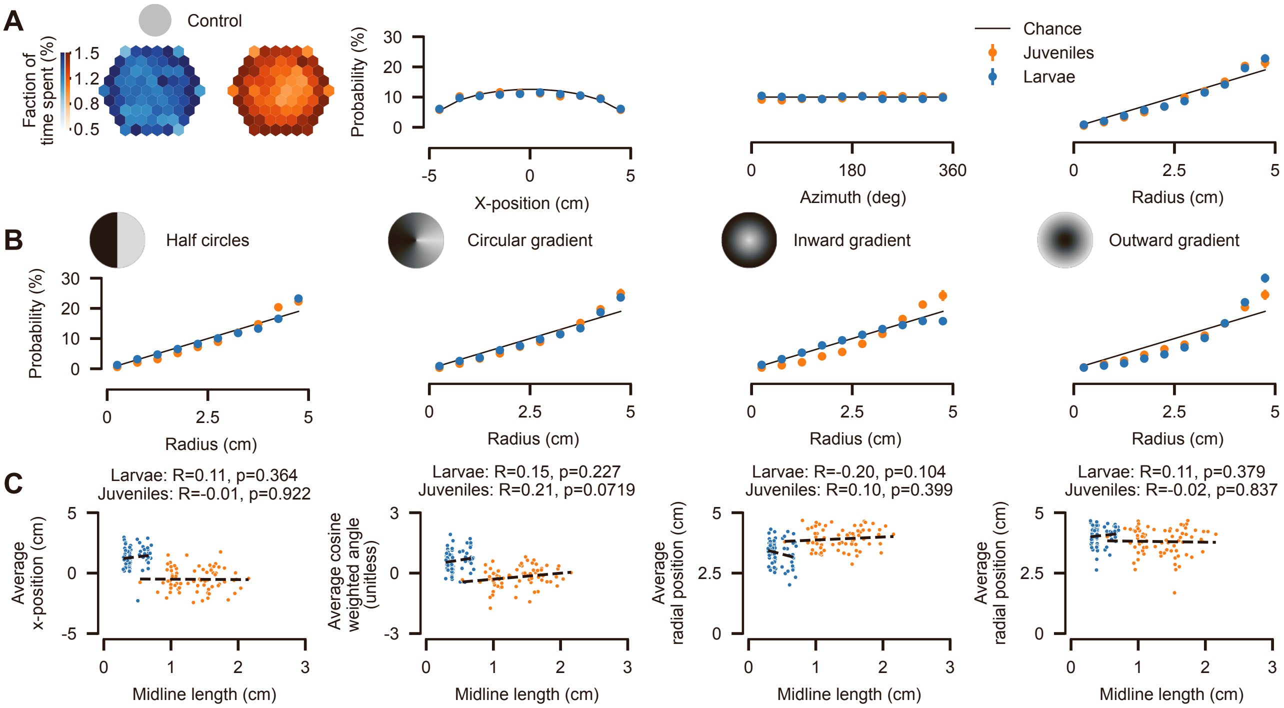

Figure S1. Controls for static luminance experiments. (A) Larvae and juveniles are distributed homogeneously in the *control stimulus* (uniform gray, 300 lux, across the arena). Fraction of time (%) spent at arena locations, binned using the same metrics as in **Figure 1G**, but not normalized to the *control stimulus*. Mean ± SEM over fish. (B) Arena occupancy as a function of the radial distance to the center for all stimulus conditions. Wall interactions add a small additional attraction towards the rim for the *half-circles stimulus* and *circular gradient stimulus*. For the *inward and outward gradient stimuli*, behavior is a superposition of wall interaction and gradient navigation. (C) The relation between body size (as measured by the length of the midline) and phototactic behavior for individual fish. Body size within each age group does not influence the position of the fish in the arena. N = 70 larvae (blue) and 73 juveniles (orange), thin black lines indicate mathematical chance levels, and black dashed lines indicate linear fits to age populations. Related to **Figure 1**.

Figure S2. Detailed quantification of behavior in the *virtual circular gradient stimulus*. (A) Area-normalized distributions of orientation changes at different radial bins in the arena. (B) Fraction of left-directed turns as a function of whole-field brightness. Annotations indicate bootstrapped significance from zero (n.s. >= 0.05). (C) A logarithmic relationship of data in **Figure 2E** captures the modulation of behavior by whole-field brightness for juveniles better (higher R-squared) than a linear relationship. As larval behavior is not strongly modulated by homogenous whole-field luminance, both functions lead to the same fitting quality. (D) Following the analysis performed previously[44], we only observe a weak, not significant effect in streak length probabilities when compared to randomly generated turns. P-values indicate statistical significance (Mann-Whitney U-test comparing randomly generated turns to real data). (E) Turn pair counting for individual fish does not show a significant difference from shuffled data either. N = 46 larvae (blue) and 30 juveniles (orange). Black vertical bars indicate 95% bootstrapped confidence intervals. Related to **Figure 2**.

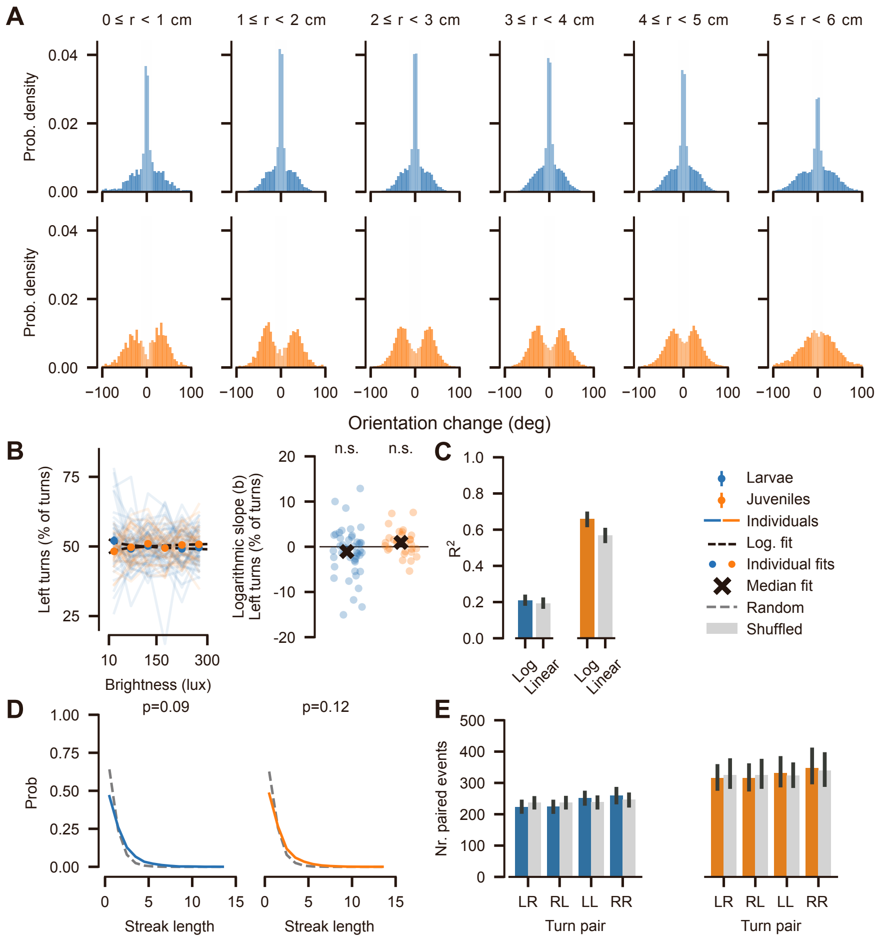

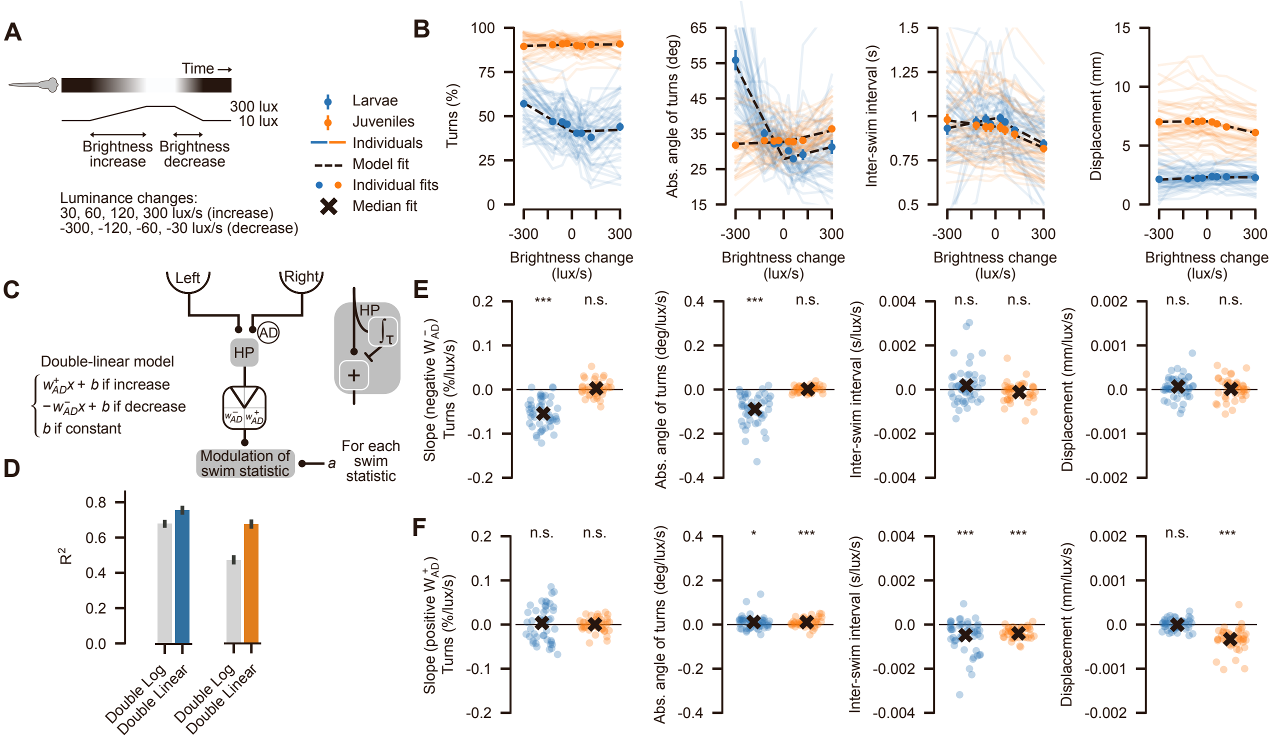

Figure S3. Whole-field luminance changes. (A) We present whole-field luminance changes by linearly increasing or decreasing the arena brightness with either 30, 60, 120, or 300 lux/s. (B) Swimming behavior as a function of temporally changing whole-field brightness levels: Percentage of turns relative to all swims, absolute angle of turns, inter-swim interval, and displacement. Mean ± SEM over fish. Thin colored lines are median values within individual fish, and the black-dashed line is the average over the fits of individual fish. (C) Averaging-Derivative pathway (AD) model. (D) A double-linear relationship (larvae, blue; juveniles, orange) captures the modulation of behavior by whole-field brightness better than a double logarithmic relationship (gray). (E, F) Fitted slopes for individual fish (circles) of the same data as in B. N = 54 larvae (blue) and 43 juveniles (orange). The black cross is the mean over individual slopes. Annotations indicate bootstrapped significance from zero (p*** < 0.005; p* < 0.05; n.s. >= 0.05; Methods). Related to **Figures 2** and **3**.

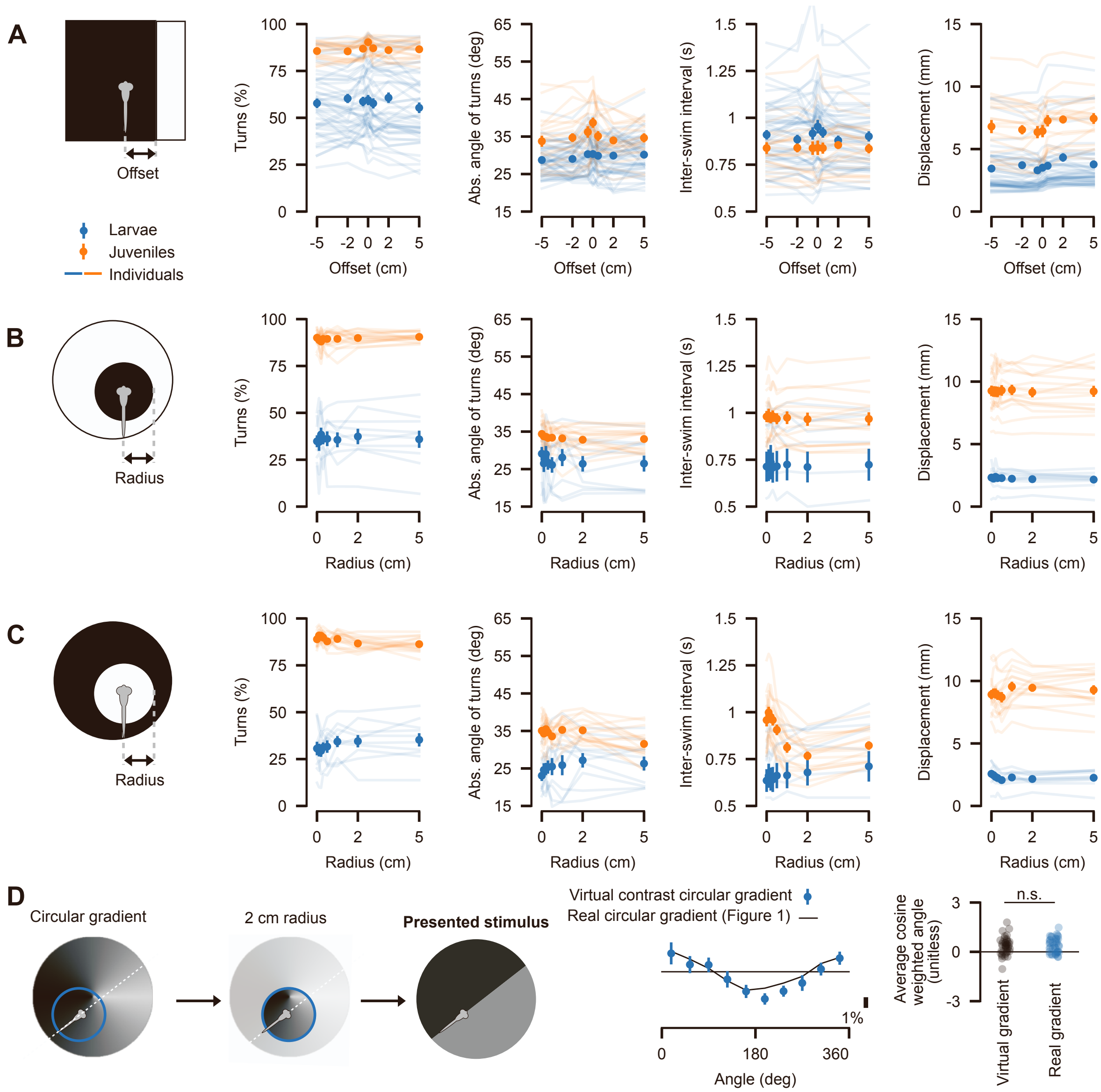

Figure S4. Field of view measurements reveal the spatial extent of luminance calculations. To gain an impression of the field of view of larval and juvenile zebrafish for luminance calculations, we quantified swim characteristics for three different fish-locked stimuli. (A) A stimulus where the lateral offset position of the contrast edge is varied relative to the midline of the fish. (B) A dark disk centered at the position of the fish in a bright environment. (C) A bright disk in a dark environment. For each configuration, we quantified how behavior is modulated by the offset or radius of the stimulus. (D) *Virtual contrast circular gradient stimulus*: Fish are shown a spatial contrast locked to the orientation and position of the fish (right). The brightness levels for the left and right sides are determined by averaging brightness values within a 2 cm radius on either lateral side (middle) of the fish coordinates with a lookup table using the *real circular gradient stimulus* (left, and **Figure 1C**). Compared to the *real circular gradient stimulus* (thin black line), luminance navigation performance is not significantly different. Mean ± SEM over individual fish, thin colored lines are medians within individual fish. A: N = 47 larvae (blue) and 20 juveniles (orange), B and C: N = 10 larvae and 16 juveniles, D: N = 22 larvae. Related to **Figure 4**.

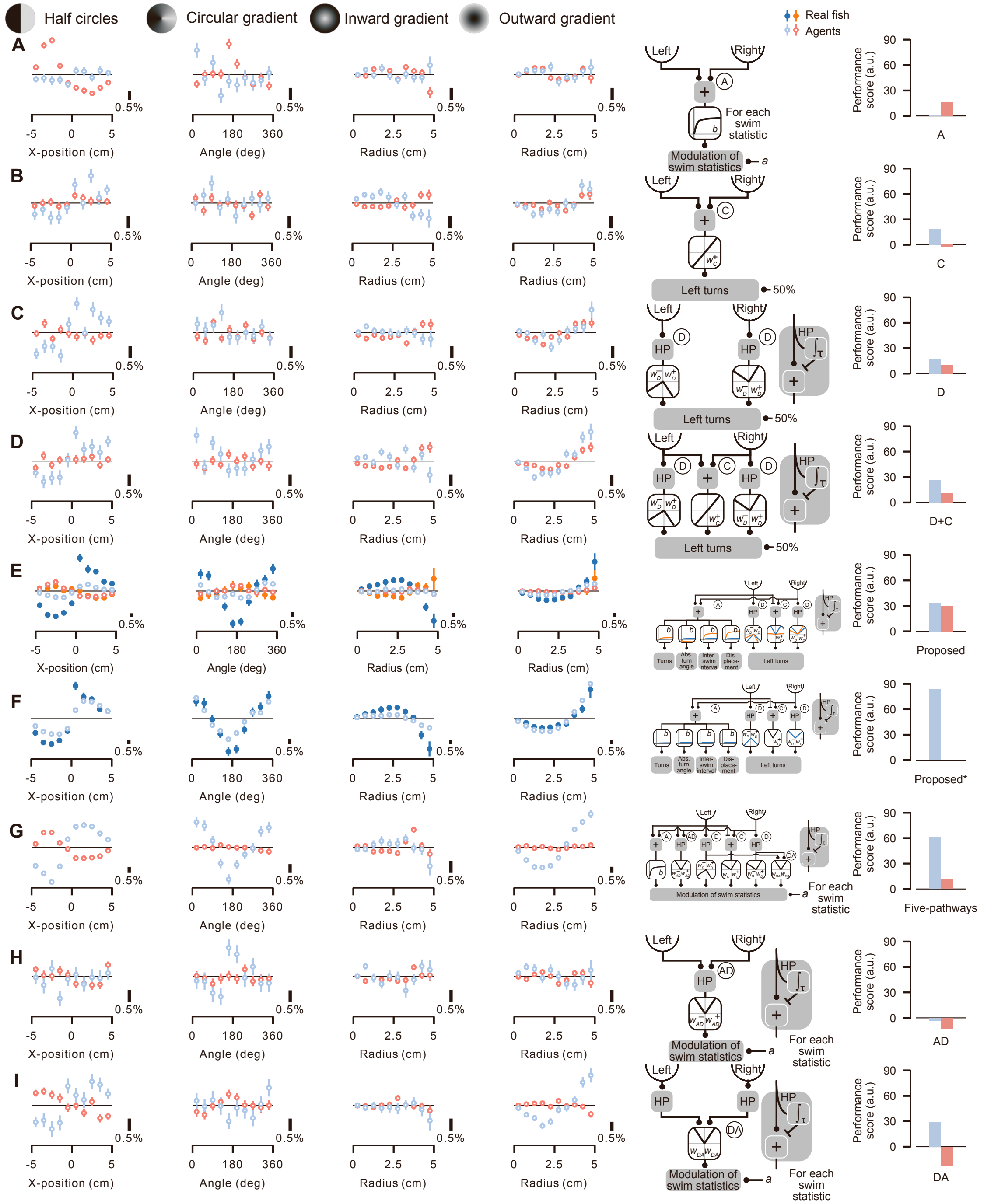

Figure S5. Detailed model predictions for all agent model variants. (A–I) First four columns: Fraction of time (%) spent at arena locations, binned using stimulus-specific metrics (see axis labels in **Figure 1C**), relative to the *control stimulus* (all gray). Mean ± SEM over agents. Fifth column: model illustration. Sixth column: Performance score. A to I: Averaging pathway model (A), Contrast pathway model (C), Derivative pathway model (D), Derivative and Contrast pathway model (D+C), the proposed model (Proposed), the proposed model (Proposed*) with manually tuned Contrast weight (larval agent only), Extended model (Five-pathways model), Averaging-Derivative pathway model (AD), and Derivative-Averaging pathway model (DA). N = 96 agents for each model simulation and age group, larval-agents (hollow light blue), juvenile-agents (hollow red), 70 real larvae (solid blue), and 72 real juveniles (solid orange). Related to **Figure 4**.

Figure S6. The extended Five-pathways model for the *lateral brightness stimulus* and the *virtual circular gradient stimulus*. (A) Swimming behavior in response to the *lateral brightness stimulus* (**Figure 3B**): Percentage of turns relative to all swims of all swims, fraction of left turns of all turns, absolute angle of turns, inter-swim interval, and displacement over time. (B) Extended model including all pathways: Averaging (A), Averaging-Derivative (AD), Derivative (D), Contrast (C), and Derivative-Averaging (DA) pathway. (C) Swimming behavior as a function of uniform brightness level during the *virtual circular gradient stimulus*. (**Figure 2A**). A: N = 45 larvae (blue) and 52 juveniles (orange), C: N = 46 larvae and 30 juveniles. Thin, solid, colored lines are individual fish. Black dashed line: mean over individual model fits. Related to **Figures 3** and **4**.

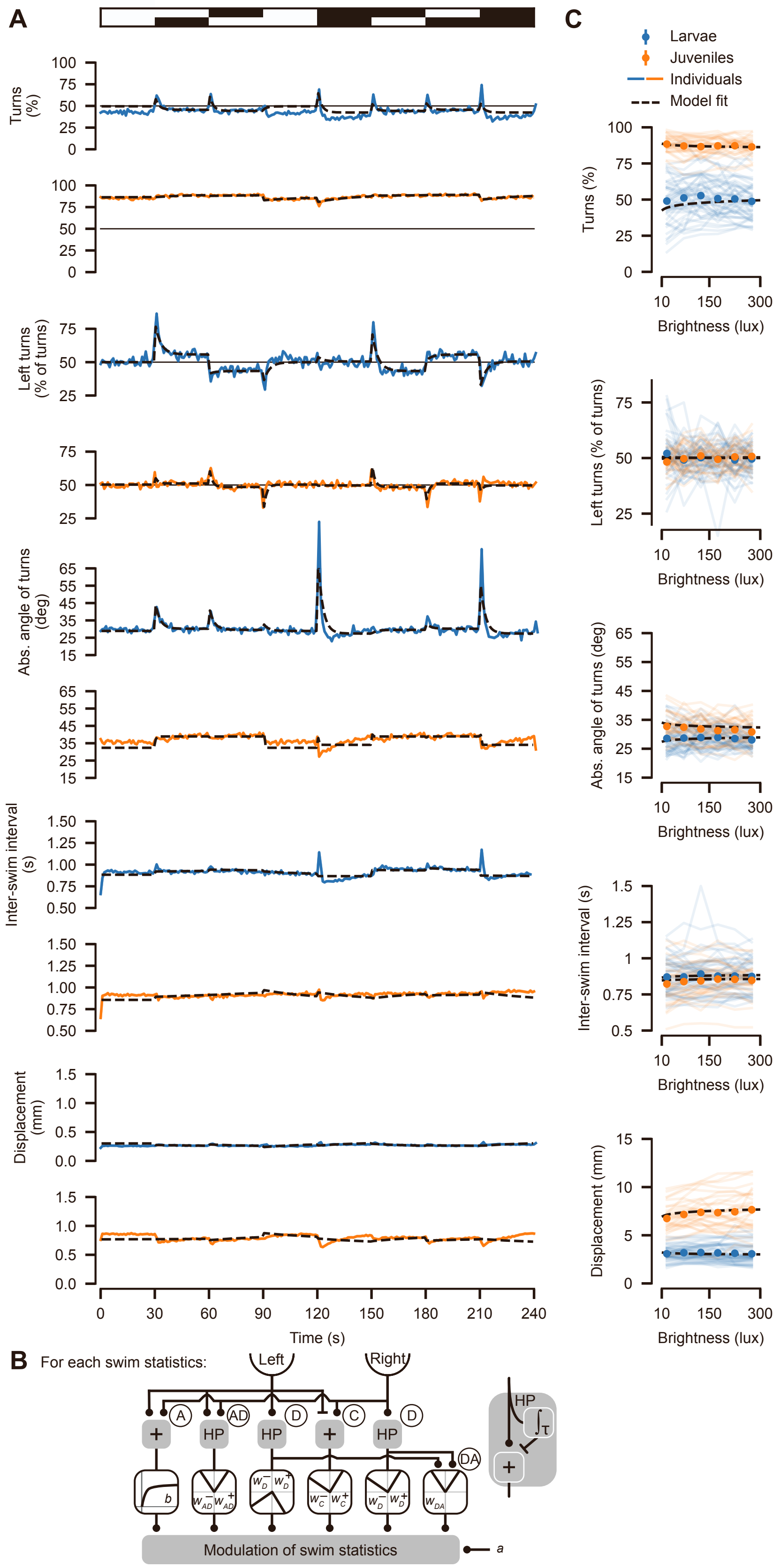

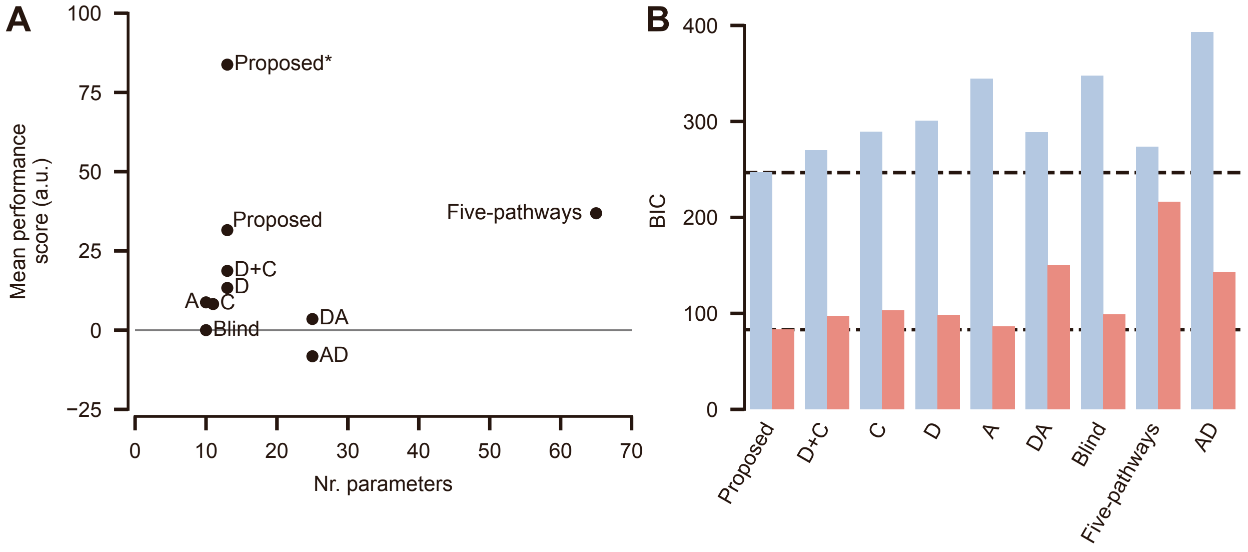

Figure S7. Simulated model performance across all tested agent configurations. (A) Mean performance scores over larval and juvenile-based models vs model parameters. Blind: Constant luminance input, C: Contrast pathway, D: Derivative pathway, D+C: Derivative and Contrast pathway, Proposed: proposed model, Proposed*: proposed model with manually tuned Contrast weight (larval agent only), DA: Derivative-Averaging pathway, AD: Averaging-Derivative pathway, Five-pathways: extended model. (B) Bayesian Information Criterion (BIC) to compare model performance given their number of parameters. For both larvae (blue) and juveniles (orange), the BIC is smallest (= best) for our proposed model. Related to **Figure 4**.
